# Application of Appropriate Use Criteria for Echocardiography in Pediatric Patients with Palpitations and Arrhythmias

**DOI:** 10.1097/pq9.0000000000000364

**Published:** 2020-10-26

**Authors:** Soham Dasgupta, Michael Kelleman, Ritu Sachdeva

**Affiliations:** From the * Division of Pediatric Cardiology, Department of Pediatrics, Children’s Healthcare of Atlanta, Emory University, Atlanta, Ga.; † Pediatrics Biostatistics Core, Emory University, Atlanta, Ga.

## Abstract

Before the integration of the pediatric appropriate use criteria (AUC) for initial transthoracic echocardiography (TTE) in the outpatient setting with our electronic medical record (EMR), there was a high proportion of “rarely appropriate” TTEs (17.2%) ordered for palpitations/arrhythmias. We studied appropriateness ratings and applicability of pediatric AUC on the initial outpatient evaluation of children with palpitations/arrhythmias after EMR integration and the yield of abnormal TTEs for these indications.

**Methods:**

We obtained data after the EMR integration of the AUC at our institution. The TTE ordering physician assigned the AUC indication and the corresponding appropriateness ratings autopopulated as: appropriate (A), may be appropriate (M), and rarely appropriate (R). We recorded the abnormal TTE findings.

**Results:**

A total of 463 TTEs were ordered for palpitations/arrhythmias. Overall, 142 (30.7%) were for A, 263 (56.8%) for M, 41 (8.8%) for R, and 17 (3.7%) for “unclassifiable” indications. Only 14 (3.0%) had abnormal TTE findings, of which none were for indications rated R. A TTE ordered for premature ventricular contractions in a 17-year-old revealed a significant abnormality (moderate atrial septal defect).

**Conclusions:**

The integration of AUC with the EMR significantly improved the appropriate utilization of TTE for palpitations and arrhythmias with a decrease in the proportion of TTEs for R indications (17.2%–8.8%). Although the yield of abnormal findings on TTE performed for palpitations/arrhythmias is quite low, the AUC ratings successfully stratified the indication with no abnormalities for indications rated R. These findings can guide future revisions of AUC indications and ratings to optimize resource utilization.

## INTRODUCTION

Palpitations and arrhythmias are a common indication for transthoracic echocardiography (TTE) ordered by outpatient pediatric cardiologists. The first pediatric appropriate use criteria (AUC) published in 2014 provides guidelines for initial outpatient evaluation of pediatric cardiology patients by TTE in the outpatient setting.^1^ Clinical scenarios related to “palpitations and arrhythmias” comprised 16 of the 113 indications in this document (AUC indications 1–16).^1^

The Pediatric Appropriate Use of Echocardiography (PAUSE) study, which was a large multicenter AUC implementation study, reported the baseline appropriateness of initial outpatient TTEs before the release of the AUC document.^2^ This study reported that TTEs ordered for palpitations, even in the setting of an abnormal electrocardiogram (ECG), had a meager yield of abnormal findings (1.4%). Additionally, selective data for our institution obtained from this study showed that there was a high proportion of TTEs ordered for AUC indications 1–16 that were “rarely appropriate” (R) (17.2%) based on the AUC document. The PAUSE study demonstrated that active educational interventions rather than a passive release of the document were critical to improve the appropriateness of TTE orders.^2–4^ In June 2017, we incorporated the AUC into our electronic medical record (EMR) as a decision support tool to improve its accessibility to the ordering physicians. This intervention led to a significant overall decrease in the proportion of TTEs ordered for indications rated R from 4.8% in the pre-EMR phase to 2% after EMR integration.^5^ Given the relatively high proportion of TTEs rated R for initial outpatient evaluation of palpitations and arrhythmias during the PAUSE study, we sought to determine the appropriateness of TTEs ordered in these patients following EMR integration. A secondary aim was to determine the yield of abnormal findings on TTEs ordered for these indications. We hypothesized that the integration of AUCs with the EMR would improve the appropriateness of TTEs ordered for these indications. Still, the yield of abnormal TTE findings will be low even for indications rated “Appropriate” (A).

## METHODS

### Study Design and Data Collection

The Institutional Review Board of the Children’s Healthcare of Atlanta approved this study. The study included all patients younger than 18 years who underwent initial outpatient evaluation at our center and had a TTE ordered by an outpatient cardiologist for AUC indications # 1–16 between June 2017 and October 2019 after AUC integration with the EMR. During the study period, the ordering physician assigned AUC indications while ordering the TTE within the EMR. The corresponding ratings [A, may be appropriate (M), R] autopopulated within the EMR. There was a provision to label an indication as “unclassifiable” and describe it if it was not available in the AUC document. The pre-EMR period consisted of 3 distinct phases of the PAUSE study. Phase I included data from 6 months before the release of the AUC document.^2^ Phase II included four months following the AUC document’s release without any educational intervention.^3^ A single reviewer (R.S.) assigned AUC indications and corresponding ratings upon review of clinic notes during these 2 phases. Phase III involved a multifaceted educational intervention for 4 months for all the clinic physicians.^4^ This included sharing of the pre-educational intervention results from the PAUSE study^2^ (individual, site, and overall study results), lectures by on-site investigators on how to use the AUC document and active audit and feedback by the site investigator. As a part of this educational intervention, during this phase, the physicians were required to select the AUC indication before ordering a TTE. The appropriateness ratings corresponding to these indications were used. Following integration with the EMR, data for appropriateness of TTE orders have been extracted from the EMR quarterly, and feedback has been provided to individual physicians regarding their appropriateness ratings and average center ratings. We reviewed medical records to obtain ECG findings and TTE results. The outpatient cardiologist interpreted the ECG findings at the time of the clinic visit. They were not reinterpreted by the investigators since the clinic physician’s interpretation of the ECG guided the decision to perform the TTE.

### Classification of TTE Findings

The TTE findings included the specific abnormality, severity, and whether it was related to the indication of ordering the TTE. The TTE findings were normal, incidental, or abnormal. Incidental findings were defined based on the previously published PAUSE study.^2^ They included patent foramen ovale, peripheral pulmonary stenosis, patent ductus arteriosus in a neonate, and left superior vena cava, retroaortic innominate vein, left arch with aberrant subclavian, common brachiocephalic trunk, and tiny coronary fistula. Also, a finding that was not thought to be related to the indication for ordering the echo was defined as incidental. Abnormal findings were mild, moderate, or severe based on the criteria used in the previously published PAUSE study^2^. We also determined if the TTE findings were related to the indication.

### Study Outcomes

The primary outcome was the proportion of TTEs ordered for indications rated R on the initial outpatient evaluation of children with palpitations and arrhythmias. The secondary outcome was the yield of abnormal findings on TTEs ordered for AUC indications # 1–16.

### Statistical Analysis

SAS 9.4 was used to perform statistical analyses (statistical significance < 0.05). We calculated descriptive statistics for all variables of interest. We used medians and ranges for continuous variables and counts and percentages for categorical variables. The proportion of R rated TTEs between the pre-EMR and the EMR phase was compared using the simple Chi-square test.

## RESULTS

**Fig. 1.**
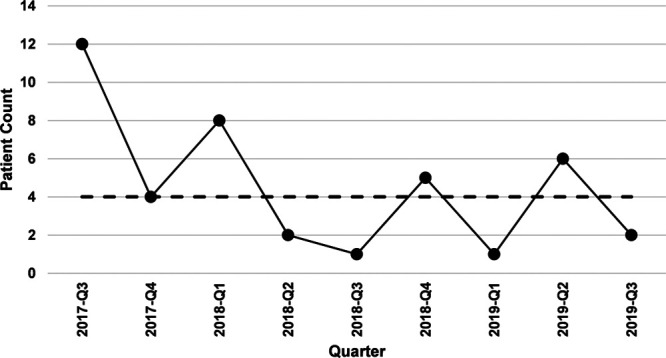
The absolute number of patients per quarter that had a TTE ordered for an indication rated R during the EMR phase (median = 4 patient count).

Of the total 19,328 TTEs performed during the study period, 463 (2.4%) were for indications related to palpitations and arrhythmias. The median age (25th–75th percentile) of patients included in the study was 13 years (9–16 years), with the majority of the studies performed in those older than 5 years old (n = 409; 88.3%). Overall, 142 (30.7%) studies were for indications rated A, 263 (56.8%) for M, 41 (8.8%) for R, and 17 (3.7%) for “unclassifiable” indications (Table 1). The proportion of TTEs performed for AUC indications rated R were 28%, 11.9%, and 11.3% in phases I, II, and III of the pre-EMR phase, respectively (17.2% over the entire pre-EMR phase). This proportion declined significantly in the EMR phase, from 17.2% to 8.8% (*P* = 0.005). Figure 1 shows the absolute number of patients per quarter that had a TTE ordered for indication rated R. The median patient count for the EMR phase was 4. Similar data were unavailable for the pre-EMR period. There was no difference in TTEs ordered for indications rated R based on the age of the patient. Supraventricular tachycardia and ventricular tachycardia (AUC indications # 11 and 14, respectively) were the 2 most common indications rated A, and palpitations with abnormal ECG (AUC indication # 3) was the most common indication rated M. Palpitations with no other signs of cardiovascular disease, an unremarkable family history, and a normal ECG (AUC indication # 2) were the most common indication rated R (Table 1).

**Table 1.** TTE Abnormalities Stratified by AUC Indications and Their Appropriateness Rating

AUC Indication Number	AUC Indication	Appropriateness Rating	TTE N	Type and N of TTE Abnormalities
1.	Palpitations with no other symptoms or signs of cardiovascular disease, a benign family history, and no recent ECG	R	3	
2.	Palpitations with no other symptoms or signs of cardiovascular disease, a benign family history, and a normal ECG	R	18	
3.	Palpitations with abnormal ECG	M	134	Mitral valve prolapse (1)
4.	Palpitations with family history of a channelopathy	R	2	
5.	Palpitations in a patient with known channelopathy	M	0	
6.	Palpitations with family history at a young age (before the age of 50 y) of sudden cardiac arrest or death and/or pacemaker or implantable defibrillator placement	A	6	
7.	Palpitations with family history of cardiomyopathy	A	6	
8.	Palpitations in a patient with known cardiomyopathy	A	0	
9.	PACs in the prenatal or neonatal period	R	1	
10.	PACs after the neonatal period	R	11	
11.	Supraventricular	A	108	Small ASD (2)
	tachycardia			Bicuspid aortic valve without stenosis or insufficiency (1)
12.	PVCs in the prenatal or neonatal period	M	2	
13.	PVCs after the neonatal period	M	127	Moderate ASD (1)
				Mitral valve prolapse (2)
				Small PDA beyond neonate (1)
				Bicuspid aortic valve without stenosis or insufficiency (1)
14.	Ventricular tachycardia	A	22	Small ASD (1)
				Mitral valve prolapse (1)
				Bicuspid aortic valve without stenosis or insufficiency (1)
15.	Sinus bradycardia	R	5	
16.	Sinus arrhythmia	R	1	

A, appropriate; ASD, atrial septal defect; AUC, appropriate use criteria; M, may be appropriate; PAC, premature atrial contraction; PDA, Patent ductus arteriosus; R, rarely appropriate.

The study investigator (S.D.) reviewed the clinical notes of patients with TTEs ordered for “unclassifiable” indications (N = 17) and determined that 12 of 17 could be classified into one of the AUC indications in the document. Other “unclassifiable” scenarios included premature junctional contractions (N = 4) and inappropriate sinus tachycardia without any palpitations (N = 1). There were only 2 unused palpitations and arrhythmia-related indications from the AUC document: “Palpitations in a patient with known channelopathy” (AUC indication # 5) and “Palpitations in a patient with known cardiomyopathy” (AUC indication # 8).

TTE findings were normal or incidental in 449 (97.0%) and abnormal in only 14 (3.0%) TTEs. None of the abnormal TTE findings were for an indication rated R (Table 2). The majority of abnormalities were minor (13/14; 92.8%), and only 1 patient (17 years old) had a significant abnormality (moderate atrial septal defect) noted on a TTE ordered for premature ventricular contractions (PVCs) (AUC indication # 13). The abnormalities were unrelated to the indication for performing the TTE. The most common indication (134/463, 29%) for performing a TTE was palpitations with an abnormal ECG (AUC indication # 3), which is M rated. For this indication, the most common ECG abnormalities were left ventricular hypertrophy, T-wave inversion, and PVCs (Table 3). Other ECG abnormalities for this indication included ventricular pre-excitation, right and left axis deviation, incomplete and complete right bundle branch block, premature atrial and junctional contractions, left atrial enlargement, and first-degree atrioventricular block. However, only 1 patient had an abnormality noted on their TTE (mitral valve prolapse).

**Table 2.** Number and Type of TTE Abnormalities Stratified by AUC Appropriateness Rating

Appropriateness Rating	Total Number (N = 463)	No. TTE Abnormalities	Type of TTE Abnormality
Appropriate	142 (30.7%)	6	Mitral valve prolapse (3)
			BAV w/o stenosis or insufficiency (2)
			Small ASD (1)
May Be appropriate	263 (56.8%)	6	Small ASD (2)
			Moderate ASD (1)
			Small PDA beyond neonate (1)
			Mitral valve prolapse (1)
			BAV w/o stenosis or insufficiency (1)
Rarely appropriate	41 (8.8%)	0	-
Unclassifiable	17 (3.7%)	2	Small VSD (1)
			BAV w/o stenosis or insufficiency (1)

ASD, atrial septal defect; BAV, bicuspid aortic valve; PDA, patent ductus arteriosus; VSD, ventricular septal defect.

**Table 3.** ECG Findings in Patients who Had a TTE Ordered for AUC Indications 1–8

AUC Indication Number	AUC Indication	Appropriateness Rating	TTE (N)	ecg abnormalities
1.	Palpitations with no other symptoms or signs of cardiovascular disease, a benign family history, and no recent ECG	R	3	RVH (1)
2.	Palpitations with no other symptoms or signs of cardiovascular disease, a benign family history, and a normal ECG	R	18	None
3.	Palpitations with abnormal ECG	M	134	LVH (23)
				PVC (20)
				T-wave abnormalities (16)
				Other (51)
4.	Palpitations with family history of a channelopathy	R	2	None
6.	Palpitations with family history at a young age (before the age of 50 y) of sudden cardiac arrest or death and/or pacemaker or implantable defibrillator placement	A	6	PAC (1)
				Incomplete RBBB (1)
				Prolonged QTc (1)
7.	Palpitations with family history of cardiomyopathy	A	6	LVH (1)
				Incomplete RBBB (1)
				PJC (1)

AUC, appropriate use criteria; LVH, left ventricular hypertrophy; PVC, premature ventricular contraction; PAC, premature atrial contraction; PJC, premature junctional contraction; RBBB, right bundle branch block; RVH, right ventricular hypertrophy.

## DISCUSSION

This study demonstrates a significant decline in the TTEs ordered for indications rated R for patients presenting with palpitations and arrhythmias following the integration of AUCs within the EMR. Although the AUC ratings successfully stratified TTEs based on no abnormalities found in those rated R, there remains a meager yield of abnormal findings even in the studies ordered for indications rated A and M.

Using the pediatric AUC, the PAUSE study demonstrated the benefits of active educational intervention and feedback in improving the appropriateness of TTEs.^2–4^ Although the PAUSE study could have influenced the decreasing proportion of rarely appropriate TTEs, the long time frame of 2 years between the end of the PAUSE study and EMR integration makes it less likely. Based on previously published studies, these effects do not seem sustainable without the AUC integration within the clinical workflow.^6,7^ Although one could argue that the providers may have a bias in choosing only the indications rated A when using AUCs integrated with the EMR, previous studies in adult patients have demonstrated the benefit of making AUCs available to the providers as a decision support tool.^8,9^ Similar to our overall results after EMR integration and studies in adults, this study also demonstrated a significant decline in TTEs ordered for indications rated R from 17.2% to 8.8% following EMR integration.^5^ During phases I and II of the PAUSE study, each chart was reviewed by the site investigator to assign the AUC indication. The idea behind the EMR integration of AUC was not only to make it readily available to the physicians but also to avoid the intensely difficult task of the manual audit of the charts and personal feedback to the ordering physician. The data from the EMR phase presented in this study reflect the real-time clinical practice where the clinic physician responsible for ordering the TTE assigned the AUC indication rather than the site investigator. Although a review of patient records could reveal a more appropriate indication, such as that related to a pathologic murmur, abnormal ECG, or exertional chest pain, we purposefully chose not to do a clinical chart review for each TTE order to reclassify the indication.

Even though palpitations and arrhythmias are common complaints leading to pediatric cardiology clinic referrals, previous studies have shown that the yield of abnormal TTEs ordered for palpitations is low. A study in pediatric patients presenting with palpitations reported that only 1 of 134 had an abnormality on TTE (atrial septal defect).^10^ Another study also reported that a TTE performed in 48 of 190 patients for this indication, had a positive finding in only one patient. A thorough history and physical examination and baseline ECG were able to identify the etiology more commonly.^11^ Consistent with other studies, our study demonstrates a low yield of abnormal TTEs ordered for these indications. The abnormal TTE findings were unrelated to the indication for ordering it (palpitations and arrhythmias). Importantly, there were no abnormalities in TTEs performed for R rated indications per the AUC document.

In this study, the most common indications for ordering a TTE were “palpitations with an abnormal ECG” (AUC indication # 3), “supraventricular tachycardia” (AUC indication # 11), and “PVCs after the neonatal period”(AUC indication #13). “Palpitations with an abnormal ECG” has been rated as M in the AUC document. We previously reported that the yield of TTEs performed for an abnormal ECG in an asymptomatic patient is low (6.5%) except for specific ECG findings suggestive of right heart disease.^12^ Our current study validates these findings with a low yield of abnormal TTEs even in the presence of abnormal ECG findings such as left ventricular hypertrophy, T-wave abnormalities, premature atrial contractions, supraventricular tachycardia, and PVCs. Ordering TTE for supraventricular tachycardia is rated A, given its association with congenital heart disease such as Ebstein’s anomaly.^13^ A recent study suggested that TTE may not be a necessary part of the initial evaluation of pediatric patients with new-onset supraventricular tachycardia, no prior heart disease, and the absence of ventricular pre-excitation on baseline ECG given the extremely low yield of abnormal findings (0.3%).^14^ Similarly, our study had a low yield of abnormal TTE findings ordered for this indication (2.7%) that were all unrelated to the indication.

The 2 indications that were unused in the current study could be eliminated from any future revisions of this document. AUC indication #5 (palpitations in a patient with known channelopathy) is redundant with AUC indication #44 in the document (known channelopathy). Both are rated M.^1^ It was not surprising that AUC indication #8 (palpitations in a patient with known cardiomyopathy) was unused because the current document is only for initial outpatient TTE evaluation. A previous TTE would have already been performed to establish the diagnosis of cardiomyopathy. Also, AUC indication #7 (palpitations in a patient with a family history of cardiomyopathy) could be eliminated from future revisions. It is redundant given the fact that AUC indications #92, 93, and 94 are rated A for family history of different forms of cardiomyopathies even in asymptomatic patients.^1^

Although the yield of TTEs ordered for palpitations and arrhythmias is low, there may be specific scenarios where there is value in performing a TTE to rule out pathology, and a negative test may be reassuring. These scenarios may include periprocedural planning for a patient undergoing ablation for arrhythmia. The utility of TTE in clearance for participation in competitive athletics and due to parental concerns remains controversial and is not included in the AUC document. Further revisions of the AUC document may consider adjustment of the indications for ordering a TTE in these settings, and, perhaps, the appropriateness ratings could be re-evaluated based on significantly low yield for several indications rated M. This may, in turn, improve healthcare resource utilization and reduce cost related to TTE.

Other commonly used diagnostic modalities for the diagnosis and management of patients with palpitations include a Holter monitor, an event monitor, and a loop recorder. In patients with palpitations, the yield of a Holter monitor in the setting of a normal ECG is reported to be low (5.7%).^15^ However, cardiac event monitors (52.2%) and loop recorders (up to 83%) may have an increased diagnostic yield compared with the Holter monitors.^16,17^ Currently, there are no pediatric AUC guidelines to guide the application of these tests. Although, in the adult, AUC document palpitations are rated as A for obtaining a TTE, this indication is grouped with other symptoms related to a suspected cardiac etiology such as chest pain, shortness of breath, and transient ischemic attack and does not apply to the pediatric population.^18^

Its retrospective nature limits our study. We were unable to capture patients that presented with palpitations or arrhythmias and did not have a TTE due to limitations in identifying such patients. Capturing these patients would have allowed us to present the actual rate of appropriateness rather than the proportion of TTE for each appropriateness level. We did not review all patients’ medical records to reassign indications except those deemed “unclassifiable.” Also, THE designation of the AUC indication depended on the ordering physician, and we did not make changes to the AUC indication based on clinical findings. Our study did not specifically explore physician characteristics related to the appropriateness of TTE orders given the low number of those rated R. Finally, the EMR does not have a provision for providing further explanation when an indication rated R is chosen.

## CONCLUSIONS

The integration of the AUC with the EMR significantly improved the appropriate utilization of TTE for palpitations and arrhythmias, with a decrease in the proportion of TTEs for indications rated R from 17.2% to 8.8%. The yield of abnormal findings on TTE for AUC indications 1–16 is low even for those rated A or M, unrelated to the indication for the TTE. Future revisions of the AUC document should take these findings into account for refining the indications and rating them.

## DISCLOSURE

The authors have no financial interest to declare in relation to the content of this article.
